# Job Autonomy and Work Meaning: Drivers of Employee Job-Crafting Behaviors in the VUCA Times

**DOI:** 10.3390/bs13060493

**Published:** 2023-06-12

**Authors:** Ting Nie, Min Tian, Mingyang Cai, Qiao Yan

**Affiliations:** Management Department, School of Business, Macao University of Science and Technology, Macao 999078, China; tnie@must.edu.mo (T.N.); 2009853GBB30025@student.must.edu.mo (M.T.); 3220002990@student.must.edu.mo (M.C.)

**Keywords:** job autonomy, work meaning, harmonious work passion, job-crafting behavior, perceived organizational change

## Abstract

In the volatile, uncertain, complex, and ambiguous environment (VUCA), employees can better match the organization and jobs by crafting their job perceptions, work tasks, and relationships, which is valuable to maintain organizational sustainable competitiveness and promote employees’ personal growth. This study explores the influence mechanisms of job autonomy and work meaning on employees’ job-crafting behaviors and the moderating effect of perceived organizational change through a survey of 318 employees in Chinese companies. The results show that job autonomy and work meaning can promote employees’ job-crafting behaviors by increasing individuals’ harmonious work passion. The indirect effects of job autonomy and work meaning on employee job-crafting behaviors through harmonious work passion are stronger for individuals with high perceived organizational change relative to those with low perceived organizational change. Organizations should concentrate on job redesign to improve employees’ job autonomy and work meaning. A climate of change should also be created within the organization to keep employees aware of the crisis. Meanwhile, employees should actively use work resources to meet the changing needs of organizational development and promote individual career development through job-crafting behaviors.

## 1. Introduction

The technological revolution, artificial intelligence, economic crisis, regional conflicts, and globalization have brought tremendous challenges to the whole society, and companies are now in an environment of volatility, uncertainty, complexity, and ambiguity, which is known as the VUCA Times [1]. In particular, the COVID-19 epidemic in early 2020 accelerated the process of global digitization, during which employees’ work styles and organizations’ operating ways also changed significantly [2]. Organizations and employees need to maintain a high level of resilience in order to respond to changing work demands and market environments. Many employees redefine and craft work content, optimize interpersonal relationships, and improve their jobs perceptions through spontaneous behaviors, which may lead to higher satisfaction and flexibility [3]. As a kind of workplace proactive behavior, job crafting has received widespread attention from managers and academics in recent years.

Many studies have confirmed the positive impact of job crafting on employees and organizations. Employees may achieve a better fit with their jobs and organizations through continuous self-adjustment, which results in improved job engagement and performance. Due to job crafting, employees will feel more motivated and well-being at work [4,5]. Organizations can maintain workforce stability, enhance their ability to cope with uncertainty, and increase innovation [6,7]. Personal traits, motivational orientation, promotion focus, task characteristics, social capital and leadership behaviors have been proven to be significantly associated with the occurrence of employee job-crafting behaviors [8,9,10,11,12,13]. However, the underlying mechanisms of these factors remain somewhat controversial, especially in today’s VUCA environment characterized by volatility, uncertainty, complexity, and ambiguity. Which is more effective in facilitating employee job-crafting behaviors, traits, environment, resources, or leaders? The context and intrinsic mechanisms of employee job crafting remain a topic that needs further refinement and in-depth exploration.

Job crafting is concerned with the individual’s initiative to redesign work in the organization, which is a bottom–up effort to change oneself at workplace [14]. According to job demand–resource theory, job resources stimulate employees’ work potential and generate high-level work engagement [15]. Job resources are the initiators of the motivational process and can significantly predict positive employee behaviors and work performance. When individuals perceive higher work control, they may have a high sense of responsibility, which can lead to a positive attitude in the face of opportunities and adjustments. Employees can gain new skills and acquire new competencies through job autonomy, which also provides opportunities for personal growth [16,17]. Work meaning can satisfy the individual’s internal needs for achievement and recognition. Individuals with high perceptions of work meaning are more likely to experience happiness from work itself, even when faced with job stresses, such as role ambiguity or role conflict [18,19]. They will have more psychological capital to cope with negative work consequences. Both job autonomy and work meaning are important positive resources in the organization and can motivate employees to work with passion. Positive emotions can enhance their abilities to cope with work demands under time or cost constraints [20], so employees with a harmonious work passion will trigger individual self-regulation, have higher initiative toward work, and will proactively adjust work arrangements. Although individuals may perceive high levels of stress, organizational change is also an opportunity for individual’s career development and personal growth. Therefore, employees with adequate work support are more likely to have a positive attitude toward change and to use their resources for continuous self-improvement [21]. They may turn work stress into work motivation and develop proactive job-crafting behaviors.

Through a questionnaire survey of 318 employees in Chinese companies, this study explores the influence mechanism of job autonomy and work meaning on employees’ job-crafting behaviors and the moderating effect of perceived organizational change. We hope to use data analysis to discuss measures and scenarios that promote employees’ job-crafting behaviors in VUCA Times. Meanwhile, we also expect that it can enrich the research about the formation and mechanism of job crafting.

In this paper, we first introduce the background and value of the study. Then, we propose the research hypotheses through a literature review. In the research design section, we introduce the procedure, sample, and measurement of this study. Next, the collected data are statistically analyzed, and the research hypotheses are verified by IBM SPSS 26, AMOS 24 (International Business Machines Corporation, Armonk, NY, USA) and Process 3.4 (software developed by Andrew F. Hayes, USA) Then, we present the theoretical and practical implications based on a discussion of the study’s findings. Finally, we conclude by pointing out the study limitations and future research directions.

## 2. Theoretical Basis and Research Hypotheses

Job autonomy is an important dimension in the job characteristics model. It refers to the extent to which employees organize their own work schedules, determine work sequence, and use their own initiative or judgment to complete their work [22]. Specifically, job autonomy involves employees’ control and decisions over work methods, work arrangements, and work standards [23], which differs from simple freedom, in that it gives employees the opportunity to make decisions at work and to have a voice in what they do [24]. Job autonomy is essentially a management practice that delegates authority and responsibility to employees so that they are thus better able to accomplish their work goals [25]. In organizations, job autonomy generally has a positive impact on employees’ work behaviors. It can satisfy employees’ intrinsic needs, help employees to gain a sense of control over their work through decision making, and increase their perceived well-being and work engagement [26,27]. Employees with high job autonomy have more opportunities to learn new knowledge or skills and experience the intrinsic value of their work. Employees with less autonomy, on the other hand, are unable to make autonomous choices about work resources and often fail to feel supported by the organization [28]. Job autonomy also promotes information exchange and knowledge sharing within the organization, which has a positive effect on employees’ personal creativity and organizational innovation [17,29].

Work meaning is a psychological state and subjective perception, which emphasizes the meaningful and valuable psychological feelings that employees experience in the workplace. It is an individual’s value judgment of work goals based on personal ideals and standards [30]. Work meaning reflects the connection between individuals and their organizations or workplaces, including commitment, loyalty, and dedication [31]. It plays a very important role in motivating employees and has a major antecedent influence on employees’ work engagement. Employees who perceive higher work meaning are more likely to spend time and effort at work and experience pleasure from their work [32]. The perceived meaning of work makes individuals value learning and growth, which can promote internal motivation. They have more energy and enthusiasm to perform challenging tasks and are usually accompanied by higher job satisfaction [33,34]. Individuals’ organizational commitment and job performance also increase as a result [35]. In addition, work meaning enables individuals to quickly rejuvenate and refocus on their work in response to stress. Employees with higher perceptions of work meaning are more prepared for and efficient with dealing with challenges and can often achieve growth in the workplace [36,37].

Employees will achieve self-transformation by changing work tasks, interpersonal relationships, and perceptions of job boundaries. This physiological and cognitive change is defined as job crafting [38]. Berg et al. [39] pointed out that job crafting is an employee change behavior to align work with their own choices, motivations, and preferences, which reflects the process of individual-initiated job redesign in the organization. It is a bottom-up proactive behavior of changing oneself in an organization rather than being pressured by external forces. Job crafting is therefore also defined as a kind of employee-initiated behavior that consists of finding resources, pursuing challenges, and reducing demands [14]. Job crafting involves both cognitive and behavioral shaping: cognitive crafting emphasizes individuals changing their knowledge and understanding of work, and behavioral crafting emphasizes individuals proactive actions to change tasks and relational work characteristics [40]. Employees balance job requirements, resources, personal abilities, and needs through continuous adjustment. Job crafting enables work to better meet employees’ unique skills, motivations, and interests [41]. Work qualifications and experiences can affect job-crafting behaviors, and individuals with rich work experiences and competencies are more inclined to work with high job control and freedom, and also develop more crafting behaviors [42,43]. In addition, individuals with proactive personalities are able to mobilize work resources and change work requirements to fit the work environment and work boundaries, which results in more job-crafting behaviors, increased work engagement, and better work performance [41]. Individuals with higher promotion-focus orientation are more sensitive to positive outcomes and are more likely to achieve satisfaction through job redesign, which also facilitates employee job-crafting behaviors [13,44].

Parker et al. [45] pointed out that autonomy motivation is the grounds and basis for employees to realize job-crafting behavior. Individuals are more receptive to change when they have autonomy in terms of work processes, timing, and methods. If an individual is able to exert some influence on change, he or she will not feel threatened and can increase their openness to change [46]. Perceived control and readiness to change can significantly influence an individual’s job-crafting behavior [47]. Employees with a high level of autonomy at work are more likely to engage in job crafting and actively explore person–job fit [48]. Work meaning can enhance an individual’s identification with the job and the organization, which can strengthen their commitment and engagement [32]. Perceived work meaning is generally accompanied by high work motivation and initiative. Employees will continually adjust work tasks, arrangements, and relationships, and this reflects more job-crafting behaviors. Research hypotheses 1 and 2 are therefore proposed:

**H1:**  *Job autonomy positively influences employees’ job-crafting behavior.*

**H2:**  *Work meaning positively influences employees’ job-crafting behavior.*

Passion is defined as a strong tendency toward personal preference, which means that an individual considers a behavior or task important and is willing to devote more material, mental, and temporal resources to that behavior or task [49]. Houlfort and Vallerand [50] introduced the concept of passion into the work context: work passion is the strong disposition or willingness of an organizational member to perform a job that the individual likes or even loves, considers important, and is ready to devote time and energy to. It is regarded as a core characteristic of self-identity. Employee work passion comes from the cognitive and emotional evaluation of work and the organization, which reflects a persistent, positive, meaningful state of well-being. Strong positive emotions are the manifestation and experience that accompany work passion development. According to the different internalization processes associated with different socio-environmental and personal factors, there are two types of workplace passions: harmonious passion (HP) and obsessive passion (OP) [50]. Harmonious passion refers to the strong motivational disposition that an individual has toward a task. Individuals with harmonious passions have the authority to choose their participation in certain activities [51]. Consequently, harmonious work passion comes from the internalization of an individual’s external motivation to engage in activity for his or her identity. When individuals are able to freely choose to participate in an activity and recognize the value of that activity, a motivational force is created that causes individuals to engage in the activity voluntarily rather than being forced to do so. Individuals can also experience a sense of pleasure and personal identity from the activity [50].

Harmonious work passion is formed through employees’ positive perceptions of their job emotions and job characteristics, which results in high well-being and increased willingness to work [52]. Social support and positive personal trait factors can also positively predict the emergence of a harmonious work passion [50]. Individuals who have harmonious work passion generate lasting, beneficial work motivation and work behaviors [53,54]. They tend to be accompanied by positive and persistent states of happiness, and consider work as a meaningful and rewarding experience [55,56]. Job autonomy and work meaning, as two positive job characteristics, satisfy the basic psychological needs of employees and can stimulate their enthusiasm and work engagement [57,58]. Positive cognitive and emotional responses from harmonious work passion can broaden employees’ thinking and actions [59]. Passionate employees autonomously internalize their work as part of their identity, and they tend to make proactive changes, such as increasing work resources and response to work challenges [55,60], which in turn generate more job-crafting behaviors. Therefore, research hypotheses 3 and 4 are proposed:

**H3:**  *Harmonious work passion mediates the relationship between job autonomy and employee job-crafting behavior.*

**H4:**  *Harmonious work passion mediates the relationship between work meaning and employee job-crafting behavior.*

Hannan and Freeman [61] defined organizational change as the process of breaking structural inertia, through which an organization changes its original structure and produces a new one to adapt to the operating environment and avoid bankruptcy. However, organizational change may not always be as disruptive as classical theories suggest. Changing the usual order, for some organizations, is a continuous growing process [62]. For employees, when organizational change occurs, there are a series of chain reactions that lead them to re-evaluate the people and events in the changing environment and to re-think future trends. The perceived change is further processed by employees to make their judgments and take the appropriate initiatives [63]. Therefore, in response to organizational change, employees seek cues from the environment to judge the meaning of the change. Employees’ attitudes and behaviors are determined by their understanding of the change situation and the impact that the change has on them [64]. The uncertainty associated with organizational change can have a negative impact on some employees and create high levels of stress. Some employees will consequently choose a cautious and conservative approach to solving problems and avoid mistakes or errors. If there is a large gap between the psychological expectation of change and reality, employee morale may be affected as a result. Employees will be more inclined to quit their jobs, and there is a significant positive relationship between employees’ perceived organizational change and their intention to leave [65]. However, it has also been shown that many employees will view organizational change as an attempt to change the status quo and an opportunity for individual development [66]. In particular, when employees believe that change can improve organizational effectiveness, they will adopt a positive attitude to support the implementation of change, thereby increasing their commitment to their work and organization [67].

Lau et al. [68] noted that when organizations implement change activities, organizational members all have specific expectations for the change and hope that managers will provide some explanations for the change. Harmonious passion reflects the deep and abiding love that individuals perceive in the workplace [69]. Employees who are harmoniously passionate about their work are always happy and excited at work, they tend to have more confidence in the future of the organization and hope that organizational change will improve efficiency and provide opportunities for personal growth, so they also tend to make proactive changes to meet the new demands. The positive effect of harmonious work passion on job-crafting behavior is stronger when the employee-perceived organizational change is higher; therefore, research hypothesis 5 is proposed.

**H5:**  *Perceived organizational change positively moderates the relationship between harmonious work passion and employee job-crafting behavior.*

In the context of organizational change, individuals are faced with changes in their work environment, work intensity, work content, and work atmosphere, which can generate a certain amount of psychological stress [70]. Job autonomy and work meaning are both resources that employees possess and can help them to identify an unfavorable external environment and respond positively to make changes. Employees with high job autonomy and work meaning tend to show more enthusiasm at work, value their jobs more, and are often willing to put in more effort at work [50]. In addition, employees who are passionate about their work are more likely to initiate personal self-regulation processes [71] and are more adaptive in the face of organizational change. Although they may experience work stress due to external factors, such as job insecurity and job uncertainty, they are more likely to turn objective stress into positive motivation. They regard them as opportunities and challenges at work, and actively change their tasks and work styles, or craft their work by learning to enhance their skills. In addition, the positive experience at work will also lead them to have better expectations for the organization’s future and to be more proactive in making changes to meet the new requirements of the company in dynamic environment. Therefore, the indirect effect of job autonomy and work meaning on job-crafting behaviors through harmonious work passion is stronger when the employee perceived organizational change is higher. Therefore, research hypotheses 6 and 7 are proposed:

**H6:**  *Perceived organizational change positively moderates the mediating effect of harmonious work passion between job autonomy and employee job-crafting behavior.*

**H7:**  *Perceived organizational change positively moderates the mediating effect of harmonious work passion between work meaning and employee job-crafting behavior.*

## 3. Method

### 3.1. Participants and Sampling Procedure

This study tries to verify the influence mechanism of job autonomy and work meaning on employee job-crafting behavior and the moderating effect of perceived organizational change through an online survey of corporate employees in China. Questionnaire surveying is a widely used method of information collection in management research because of its convenience and efficiency [72,73]. Before the survey, all respondents were informed of the purpose and procedures of the study. They were assured that all data from the study would be used for academic purposes only. Respondents completed the survey anonymously and were free to terminate answering the questionnaire at any time, and all incomplete questionnaires were considered invalid. This study was ethically reviewed by the Research Review Board of the Macau University of Science and Technology. All methods in the study were performed in accordance with the Declaration of Helsinki.

The survey was conducted online through Wenjuanxing.com, a major online survey platform in China. Most of the participants were from the Guangdong, Jiangsu, Hubei, Hunai, Guizhou, and Guangxi provinces of China. To reduce the effect of common method variance, data in this study were collected at one-month intervals. Time1:questionnaires were collected through convenience sampling and were used to measure job autonomy, work meaning, and perceived organizational change. A total of 500 questionnaires were distributed, and 441 valid questionnaires were returned, with a valid return rate of 88.2%. Time2: questionnaires were distributed to the same group of participants to measure harmonious work passion, job-crafting behavior, and demographic characteristics. A total of 441 questionnaires were distributed, and 318 valid questionnaires were returned, with a valid return rate of 72.1%.

The demographic characteristics of the respondents in this study are as follows: In terms of gender, males accounted for 49.1% (156) of respondents, females accounted for 50.9% (162) of respondents, and the gender ratio was relatively balanced. In terms of age, employees less 25 years old accounted for 5.7% (18) of respondents, employees between 26 to 30 years old accounted for 21.7% (69) of respondents, employees between 31 to 35 years old accounted for 23.6% (75) of respondents, employees between 36 to 40 years old accounted for 19.2% (61) of respondents, employees between 41 to 45 years old accounted for 13.8% (44) of respondents, employees between 46 to 50 years old accounted for 7.2% (23) of respondents, and employees more than 50 years old accounted for 8.8% (28) of respondents. The age distribution of respondents was relatively balanced. The majority of respondents were knowledge workers, and 75.2% (239) of respondents had bachelor’s degrees or higher. In terms of marital status, 73% (232) of the respondents were married. Respondents came from a wide range of industries, including manufacturing (24.2%), services (19.5%), information technology (15.4%), finance (6.3%), and other industries.

### 3.2. Measures

Self-report scales were used in the study and measured with Likert 5-point scales from “complete disagreement” to “complete agreement”. Spss 26, Amos24, and Process 3.4 were used for data analysis.

#### 3.2.1. Job Autonomy

Job autonomy involves the extent to which employees control and decide about work methods, work arrangements, and work standards [23]. Job authority was measured with a 7-item scale developed by Kirmeyer and Shirom [74], e.g., “I have the freedom to decide how to do my job”. In this study, Cronbach’s alpha coefficient of this scale was 0.862.

#### 3.2.2. Work Meaning

Work meaning is a psychological state and subjective perception, which emphasizes the meaningful and valuable psychological feelings that employees experience in the workplace [30]. Work meaning was measured with a 6-item scale developed by Bunderson and Thompson [75], e.g., “Sometimes I feel that I am destined to do this work”. In this study, Cronbach’s alpha coefficient of this scale was 0.828.

#### 3.2.3. Harmonious Work Passion

Harmonious passion is the strong motivational disposition that an individual has toward activities at the workplace [51]. Harmonious passion was measured with a 7-item scale developed by Vallerand et al. [76], e.g., “My work has given me a rich experience”. In this study, Cronbach’s alpha coefficient of this scale was 0.882.

#### 3.2.4. Job-Crafting Behavior

Job-crafting behavior is an employee-change behavior to align work with their own choices, motivations, and preferences, which reflects the process of individual-initiated job redesign in organization [77]. Job-crafting behavior was measured with a 13-item scale developed by Petrou et al. [78], e.g., “I ask others to provide feedback on my performance”. In this study, Cronbach’s alpha coefficient of this scale was 0.872.

#### 3.2.5. Perceived Organizational Change

Perceived organizational change is the employee’s perception of changes in the organization in response to internal and external environments, through which the organization can adapt to the new operating system and avoid bankruptcy [61]. Perceived organizational change was measured with a 13-item scale by Rafferty and Griffin [79], e.g., “Change often happens in my organization”. In this study, Cronbach’s alpha coefficient of this scale was 0.893.

#### 3.2.6. Control Variables

Gender, age, education, and industry were considered as control variables. The classification is as follows: gender (male and female); education (middle school, vocational school, bachelor, master and doctor); industry (manufacturing, services, information technology, finance, education, and other industries).

## 4. Results

### 4.1. Confirmatory Factor Analysis

Confirmatory factor analysis (CFA) was used to test the instrument’s validity. The result shows that the model fit of the five-factor model (job autonomy, work meaning, harmonious work passion, job-crafting behavior, perceived organizational change) is acceptable ($\chi^{2}$/*df* = 1.473, NFI = 0.903, CFI = 0.966, IFI = 0.967, RMSEA = 0.038, and RMR = 0.040), which is better than alternative models. The model fit of the single-factor model is far from acceptable ($\chi^{2}$/*df* = 6.807, NFI = 0.507, CFI = 0.544, IFI = 0.546, RMSEA = 0.133, and RMR = 0.104). In addition, the common method deviation in the study is not a big concern. In the composite reliability test, the CR values of the study variables range from 0.864 to 0.941 and are all greater than 0.8, which means that all the variables have good composite reliability. In the convergent validity test, the AVE values of the study variables ranged from 0.507 to 0.729 and are all greater than 0.5, which means that all the variables have good convergent validity.

### 4.2. Descriptive Statistics and Correlation Analysis

The results of descriptive statistics and correlation analysis are shown in Table 1. When controlling the effects of gender, age, education, and industry, job autonomy is significantly associated with harmonious work passion (r = 0.508, *p* < 0.01), and job-crafting behavior (r = 0.435, *p* < 0.01). Work meaning is significantly associated with harmonious work passion (r = 0.484, *p* < 0.01), and job-crafting behavior (r = 0.390, *p* < 0.01). Harmonious work passion is significantly associated with job-crafting behavior (r = 0.679, *p* < 0.01). In addition, perceived organizational change is significantly associated with job autonomy (r = 0.373, *p* < 0.01), work meaning (r = 0.566, *p* < 0.01), harmonious work passion (r = 0.448, *p* < 0.01), and job-crafting behavior (r = 0.428, *p* < 0.01). The preliminary correlation analysis results support the research hypotheses.

### 4.3. Hypothesis Testing

As shown in Table 2, when controlling the effects of gender, age, education, and industry, job autonomy shows a positive relationship with job-crafting behavior in model 4 (0.411, *p* < 0.001), and work meaning shows a positive relationship with job-crafting behavior in model 6 (0.389, *p* < 0.001). Therefore, Hypotheses 1 and 2 are supported.

Model 1, Model 4, and Model 5 validate the mediating effect of harmonious work passion between job autonomy and employee job-crafting behavior with the approach suggested by Baron and Kenny [80]. When controlling the effects of gender, age, education, and industry, job autonomy has a significant positive relationship with harmonious work passion (0.492, *p* < 0.001) and job-crafting behavior (0.411, *p* < 0.001). When simultaneously considering the effects of the mediating and independent variables on the dependent variable, harmonious work passion has a significant positive relationship with job-crafting behavior (0.612, *p* < 0.001). The influence of job autonomy on job-crafting behavior is decreased (0.111, *p* < 0.05). Therefore, the hierarchical regression analysis results suggest that the effect of job autonomy on job-crafting behavior is partially mediated by harmonious work passion. Thus, Hypothesis 3 is supported. Model 2, Model 6, and Model 7 validate the mediating effect of harmonious work passion between work meaning and employee job-crafting behavior. When controlling the effects of gender, age, education, and industry, work meaning has a significant positive relationship with harmonious work passion (0.486, *p* < 0.001) and job-crafting behavior (0.389, *p* < 0.001). When simultaneously considering the effects of the mediating and independent variables on the dependent variable, harmonious work passion has a significant positive relationship with job-crafting behavior (0.622, *p* < 0.001). The influence of work meaning on job-crafting behavior is not significant (0.087, *p* > 0.05). Therefore, the hierarchical regression analysis results suggest that the effect of work meaning on job-crafting behavior is fully mediated by harmonious work passion. Thus, Hypothesis 4 is supported.

Model 8 validates the moderating effect of perceived organizational change between harmonious work passion and job-crafting behavior. The hypothesis is tested by hierarchical regression analysis, in which the interaction term is introduced with the control variables and the main effects. As shown in Table 2, the moderating effect of perceived organizational change between harmonious work passion and job-crafting behavior is significant (0.110, *p* < 0.01). When employees perceive a higher level of organizational change, the impact of harmonious work passion on employee job-crafting behavior is stronger. Thus, Hypothesis 5 is supported.

The results of the moderated mediating effect validation are shown in Table 3. The 95% confidence interval of moderated mediating effect (0.089, 0.031) is [0.032, 0.154], and the interval tested in bootstrapping excludes zero, which means perceived organizational change positively moderates the relationship between job autonomy and employee job-crafting behavior through harmonious work passion. At a low level of perceived organizational change, the indirect effect of harmonious work passion between job autonomy and employee job-crafting behavior is significant (0.253,0.042), and the 95% confidence interval excludes zero [0.172, 0.337]. At a high level of perceived organizational change, the indirect effect of harmonious work passion between job autonomy and employee job-crafting behavior is also significant (0.354,0.050), and the 95% confidence interval excludes zero [0.259, 0.456]. When employees perceive a higher level of organizational change, the impact of job autonomy on employee job-crafting behavior through harmonious work passion is more significant. Thus, Hypothesis 6 is supported.

The 95% confidence interval of moderated mediating effect (0.130, 0.066) is [0.268, 0.010], and the interval tested in bootstrapping excludes zero, which means perceived organizational change positively moderates the relationship between work meaning and employee job-crafting behavior through harmonious work passion. At a low level of perceived organizational change, the indirect effect of harmonious work passion between work meaning and employee job-crafting behavior is significant (0.389, 0.061), and the 95% confidence interval excludes zero [0.270, 0.509]. At a high level of perceived organizational change, the indirect effect of harmonious work passion between work meaning and employee job-crafting behavior is also significant (0.540, 0.073), and the 95% confidence interval excludes zero [0.402, 0.687]. When employees perceive a higher level of organizational change, the impact of work meaning on employee job-crafting behavior through harmonious work passion is more significant. Thus, Hypothesis 7 is supported.

## 5. Conclusions and Discussion

### 5.1. Theoretical Implications

In the era of rapid change, it is important for enterprises’ long-term development to redesign work processes and systems that satisfy employee needs, and to stimulate their harmonious work passion to engage in job-crafting behaviors [40]. This study explores the influence mechanism of job autonomy and work meaning on employees’ job-crafting behaviors and verifies the moderating effect of perceived organizational change through the survey of employees in Chinese companies. The results show the following: Harmonious work passion mediates the relationship between job autonomy, work meaning, and employees’ job-crafting behaviors. Job autonomy and work meaning can promote employees’ job-crafting behaviors by increasing individuals’ harmonious work passion. Perceived organizational change positively moderates the relationship between harmonious work passion and employee job-crafting behaviors. Additionally, perceived organizational change positively moderates the mediating effect of harmonious work passion between job autonomy and work meaning on employee job-crafting behaviors. The indirect effects of job autonomy and work meaning on employee job-crafting behaviors through harmonious work passion are stronger for individuals with high perceived organizational change relative to those with low perceived organizational change.

This study’s findings again validate that positive work characteristics and resources are helpful for individuals to demonstrate autonomous regulation and improved behaviors [81]. Job autonomy as a management practice allows employees to make decisions about their own work by delegating authority and responsibility so that they can better accomplish their work goals [25]. Job autonomy satisfies employees’ intrinsic needs, such as gaining a sense of control and achievement, which can keep employees motivated and enthusiastic at work [26]. Work meaning not only provides the goal of work but also provides values and standards, through which employees can make judgments about their own behaviors. It can stimulate motivation and positive work attitudes. The perceived meaning of work can relieve work pressure, and enable employees to reduce helplessness and confusion at work [82]. They will have more energy to pursue growth in the workplace. Self-determination theory states that the satisfaction of individual’s basic needs for autonomy, competence, and relationships are important factors in the generation of proactive behaviors [83]. To a certain extent, if the workplace meets the individuals’ needs, they are more likely to internalize autonomy in their personal identity and thus experience a harmonious passion. Passion in the workplace comes from the cognitive and emotional evaluation of the job or organization. When employees are in a work environment with high job autonomy and meaningful work, they are prone to a positive psychological state as well as a harmonious passion for work [58]. Passionate employees view these job characteristics as positive job resources, and they tend to make proactive changes, such as increasing job resources and accepting job challenges [55,60], which can generate more job-crafting behaviors. Thus, job autonomy and work meaning can promote employees’ job-crafting behaviors by increasing individuals’ harmonious work passion.

Organizational change can bring psychological uncertainty and job insecurity to employees, and it requires their physical and psychological continuity of effort [84]. When employees perceive a higher level of organizational change, it is generally accompanied by high psychological stress. However, it does not mean that individuals are bound to resist change. When employees believe that change can improve organizational efficiency and promote personal growth, they will adopt a positive attitude to support the implementation of change, which will increase work enthusiasm and organizational loyalty [85]. This study also further validates the positive effects of perceived organizational change, especially in a volatile, uncertain, complex, and ambiguous environment. Organizational change is a transformation of the status quo and a break in organizational routine. Individuals need to constantly adapt to changing requirements to reduce the likelihood of being eliminated from the organization [86]. At the same time, change provides new opportunities for individual development. When employees have favorable resources, they are more likely to play an active role in the changing environment. They are usually motivated to learn new skills and put ideas into action to improve their work performance. Thus, under highly perceived organizational change, positive work characteristics increase individuals’ confidence to change their status quo, which enhances their enthusiasm and willingness to craft their work.

### 5.2. Practical Implications

In the VUCA world, organizational changes occur frequently and require employees to respond to the uncertainty in the working environment in a more proactive manner [1]. When an organization changes, employees can cope and respond by seeking resources, changing working ways, and critically solving problems. Job-crafting behaviors can improve employees’ ability to adapt to the dynamic environment, accelerate familiarity with new tasks, build new collaborative relationships, etc. It not only helps the organization to improve its responsiveness to the VUCA environment and market information but also promotes the career development of employees. Therefore, job crafting is a win–win initiative that benefits both the organizations and their employees.

From an organizational perspective, organizations should concentrate on job redesign to improve employees’ job autonomy and work meaning. Favorable working characteristics are the prerequisite basis for motivating individuals to make positive changes. Especially for knowledge workers, they pursue independence and their personal values, and are more willing to choose challenging jobs than mechanical and repetitive tasks. Their intrinsic need for self-control can be satisfied if they have more autonomy in terms of work processes, methods, tasks, etc. In addition, with the continuous development of the times, working has become more and more important to people in terms of its meaning and value. A job is no longer only regarded as a way to obtain financial rewards, but also as an approach to realize one’s self-worth, express one’s own assertion, and enhance one’s well-being. When assigning work tasks, companies should take into account employees’ personal preferences and let them perform the work that they like, if possible. At the same time, through in-depth communication, job value and importance can be transmitted to employees to improve their recognition of the existing work. Furthermore, organizations can create a climate of change. In the VUCA era, organizations face an unstable, uncertain, complex, and ambiguous market environment, where organizational change is the norm for survival. Organizations need to make their employees aware of the crisis and emphasize the importance and necessity of change. While some employees may be resistant to change and refuse to make adjustments, a climate of change can help identify relatively rigid employees and help them change their attitudes to accept change. Meanwhile, through the cultivation of crisis awareness, employees are encouraged to proactively use advantageous work resources to accomplish job crafting under a highly competitive and uncertain environment, which not only helps individual employees to cope with changes but also helps the organization to form strategic advantages.

From the employee’s perspective, the organization’s resources should be actively utilized to accomplish job crafting. Today’s era is dominated by high competition and uncertainty, and the COVID-19 epidemic has led to many changes in the workplace. New requirements for job tasks and skills have accelerated the elimination of traditional work patterns. It requires employees to be prepared with more diverse skills and to take the initiative to creatively respond to challenges. Employees need to learn to adapt and adjust to the rapid changes in the environment. In order to ensure job security, employees should make full use of the available resources currently, and constantly adapt to maintain a high level match with their organization and job. Individuals need to strive to be highly resilient and valuable employees [87]. In addition, job crafting can also provide individuals with opportunities for career advancement. Employees can derive greater satisfaction from their jobs by changing their perceptions and understanding of their jobs. They can also take proactive actions to change work tasks and relationships, which can assist individuals in gaining more success at work. So, job crafting not only meets organizational expectations for employees in times of VUCA, but is also an effective means for employees to cope with high uncertainty and competition at the workplace.

From the community’s perspective, when employees perceive passion about their work because of job autonomy and meaning, they are willing to make positive changes to meet the requirements of the organization through job crafting. At the same time, employees will also experience career satisfaction and organizational belonging in the process. This is a virtuous circle, which not only helps the organization retain qualified employees but also reduces the local unemployment rate and maintains social stability. In addition, employees can bring positive feelings in the workplace back to their families. Apart from providing financial support to family members, employees can share with them their gains and achievements in the workplace [88]. This helps to maintain a harmonious family relationship and to gain more understanding from family members, which can reflect the mutual facilitation of family and work.

## 6. Limitations and Future Study

There are some limitations in this study. In terms of sample characteristics, the respondents in this study are mainly knowledge employees. Their work is characterized by complexity, creativity, and challenge. They are also more sensitive to the competitive environment and have higher expectations for their career development and personal growth. Knowledge employees are more willing to turn work pressure into work motivation, and take the initiative to respond to change. Both intention and desirability of job crafting are more significant in this group. Therefore, there may be external validity issues in this study, and further validation is needed to extend the findings to non-knowledge workers. Secondly, due to the constraints of research resources, self-report scales were used in this study. The effect of common method variance was tested with a one-factor confirmatory factor analysis, which can also have an impact on the study findings. Finally, in this study, job-crafting behavior is explained only from the perspective of job autonomy and work meaning, but as an initiative of individual change in self-perception and behaviors under organizational internal and external environment, the formatting mechanism of job crafting is complex. Environmental characteristics, organizational characteristics, leading characteristics, and employee characteristics should all have some influence and can be further discussed in future studies.

Responding to the challenges of VUCA environment is essential to the survival of organizations and employees. Over the past decade, job crafting research has gained widespread attention in academia. Organizations are also constantly attempting to promote proactive adaptation among employees with positive work resources, help them cope with the work pressure from the environment, and enhance their sense of accomplishment. Promoting active and positive job crafting is of great value to the employees as well as their companies, which are win–win management practices. Future research is needed to further explore the antecedents, consequences, and boundary conditions of job crafting in order to better support employees and meet the challenges of the VUCA Times.

## Figures and Tables

**Table 1:** Mean, standard deviation and correlation statistics (*n* = 318).

	Mean	SD	1	2	3	4	5	6	7	8	9
Gender	1.51	0.501									
Age	3.71	1.660	−0.150 **								
Edu	3.17	1.134	−0.039	0.179 **							
Indu	4.89	2.893	0.093	−0.022	−0.206 **						
JA	3.36	0.689	−0.124 *	0.242 **	0.135 *	−0.078	(0.718)				
WM	3.53	0.463	−0.185 **	0.052	0.088	−0.108	0.338 **	(0.753)			
HWP	3.58	0.684	−0.064	0.185 **	0.092	−0.022	0.508 **	0.484 **	(0.728)		
JCB	3.60	0.767	−0.047	0.191 **	0.121 *	−0.014	0.435 **	0.390 **	0.679 **	(0.712)	
POC	3.60	0.567	−0.195 **	0.125 *	0.253 **	−0.047	0.373 **	0.566 **	0.448 **	0.428 **	(0.854)

Note: ** *p* < 0.01, * *p* < 0.05; JA: job autonomy, WM: work meaning; HWP: harmonious work passion; JCB: job-crafting behavior; POC: perceived organizational change.

**Table 2:** Hierarchical regression test of mediating effect (*n* = 318).

		Harmonious Work Passion		Job-Crafting Behavior					
		M1	M2	M3	M4	M5	M6	M7	M8
**Control varaibale**									
Gender		0.005	0.048	−0.018	0.016	0.013	0.049	0.019	0.030
Age		0.063	0.162 **	0.172 **	0.084	0.045	0.167 **	0.066	0.063
Edu		0.019	0.029	0.092	0.058	0.046	0.068	0.049	0.019
Indu		0.021	0.035	0.011	0.030	0.018	0.041	0.019	0.017
**Independent variable**									
JA		0.492 ***			0.411 ***	0.111 *			
WM			0.486 ***				0.389 ***	0.087	
**Mediating variable**									
HWP						0.612 ***		0.622 ***	0.591 ***
**Moderating variable**									
POC									0.169 ***
HWP X POC									0.110 **
$R^{2}$		0.262	0.266	0.045	0.201	0.477	0.189	0.474	0.497
F		22.182 ***	18.804 ***	3.655 *	15.685 ***	47.238 ***	14.546 ***	46.643 ***	43.757 ***

Note: *** *p* < 0.001, ** *p* < 0.01, * *p* < 0.05; JA: job autonomy, WM: work meaning; HWP: harmonious work passion; JCB: job-crafting behavior; POC: perceived organizational change.

**Table 3:** Moderated mediating effect (*n* = 318).

Variable	Moderating Effect				Moderated Mediating Effect						
		**Effect**	**SE**	***p***	**LLCI**	**ULCI**		**Index**	**SE**	**LLCI**	**ULCI**
	Int_1	0.181	0.066	0.006	0.052	0.311		0.089	0.031	0.032	0.154
JA	L	0.518	0.069	0.000	0.382	0.655	L	0.253	0.042	0.172	0.337
	H	0.724	0.065	0.000	0.597	0.851	H	0.354	0.050	0.259	0.456
	Int_1	0.186	0.067	0.006	0.054	0.317		0.130	0.066	0.268	0.010
WM	L	0.542	0.070	0.000	0.405	0.679	L	0.389	0.061	0.270	0.509
	H	0.753	0.063	0.000	0.629	0.877	H	0.540	0.073	0.402	0.687

Note: JA: job autonomy; WM: work meaning; LLCI: low level of 95% confidence interval; ULCI: upper level of 95% confidence interval.

## Data Availability

The data presented in this study are available on request from the corresponding author.

## Abbreviations

The following abbreviations are used in this manuscript:

JA	Job Autonomy
WM	Work Meaning
HWP	Harmonious Work Passion
JCB	Job-Crafting Behavior
POC	Perceived Organizational Change
VUCA	Volatility, Uncertainty, Complexity, and Ambiguity

## References

[1] Horney N., Pasmore B., O’Shea T. (2010). Leadership agility: A business imperative for a VUCA world. Hum. Resour. Plan..

[2] Bach C., Sulíková R. (2021). Leadership in the Context of a NewWorld: Digital Leadership and Industry 4.0. Manag. Glob. Transit..

[3] Roczniewska M., Rogala A., Marszałek M., Hasson H., Bakker A.B., von Thiele Schwarz U. (2023). Job crafting interventions: What works, for whom, why, and in which contexts? Research protocol for a systematic review with coincidence analysis. Syst. Rev..

[4] Gordon H.J., Demerouti E., Le Blanc P.M., Bipp T. (2015). Job crafting and performance of Dutch and American health care professionals. J. Pers. Psychol..

[5] Van Wingerden J., Bakker A.B., Derks D. (2017). Fostering employee well-being via a job crafting intervention. J. Vocat. Behav..

[6] Petrou P., Demerouti E., Schaufeli W.B. (2015). Job Crafting in Changing Organizations: Antecedents and Implications for Exhaustion and Performance. J. Occup. Health Psychol..

[7] Xu X.V., Jiang L., Wang H.-J. (2019). How to build your team for innovation? A cross-level mediation model of team personality, team climate for innovation, creativity, and job crafting. J. Occup. Organ. Psychol..

[8] Thun S., Bakker A.B. (2018). Empowering leadership and job crafting: The role of employee optimism. Stress Health.

[9] Kim M., Baek S.I., Shin Y. (2019). The Effect of the Congruence between Job Characteristics and Personality on Job Crafting. Multidiscip. Digit. Publ. Inst..

[10] Bajaba S.M., Alajhar N.A., Bajaba A.M. (2021). The Bottom-Up Impact of Proactive Personality on Employee Job Crafting: A Serial Mediation Model. J. Psychol..

[11] Shin I., Jung H. (2019). Differential roles of self-determined motivations in describing job crafting behavior and organizational change commitment. Curr. Psychol..

[12] Tajpour M., Salamzadeh A., Salamzadeh Y., Braga V. (2022). Investigating social capital, trust and commitment in family business: Case of media firms. J. Fam. Bus. Manag..

[13] Chen W., Xiao Y., Liu Y., Wang B. (2022). The relationship of employees’ promotion focus and job crafting: Psychological empowerment as a mediator. Soc. Behav. Personal. Int. J..

[14] Petrou P., Demerouti E., Xanthopoulou D. (2017). Regular versus cutback-related change: The role of employee job crafting in organizational change contexts of different nature. Int. J. Stress Manag..

[15] Bakker A., Tims M., Derks D. (2012). Proactive personality and job performance: The role of job crafting and work engagement. Hum. Relat..

[16] Zhou Q., Li Q., Gong S. (2019). How job autonomy promotes employee’s sustainable development? A moderated mediation model. Sustainability.

[17] Jankelová N. (2022). Entrepreneurial Orientation, Trust, Job Autonomy and Team Connectivity: Implications for Organizational Innovativeness. Eng. Econ..

[18] Salamone A., Lordan G. (2022). Can meaning make cents? Making the meaning of work salient for US manufacturing workers. PLoS ONE.

[19] Lavy S. (2022). A meaningful boost: Effects of teachers’ sense of meaning at work on their engagement, burnout, and stress. AERA Open.

[20] Talpur A.A., Awan M.S., Hashmi F., Jamal A. (2013). Evaluation & management of patients with liver trauma. Med. Channel.

[21] Gigliotti R., Vardaman J., Marshall D.R., Gonzalez K. (2019). The role of perceived organizational support in individual change readiness. J. Chang. Manag..

[22] Hackman R., Oldham R. (1976). Motivation through the design of work: Test of a theory. Organ. Behav. Hum. Perform..

[23] Breaugh J.A. (1985). The measurement of work autonomy. Hum. Relat..

[24] Cohen-Meitar R., Carmeli A., Waldman A. (2009). Linking meaningfulness in the workplace to employee creativity: The intervening role of organizational identification and positive psychological experiences. Creat. Res. J..

[25] Leach D.J., Wall T.D., Jackson P.R. (2003). The effect of empowerment on job knowledge: An empirical test involving operators of complex technology. J. Occup. Organ. Psychol..

[26] Den Hartog D.N., Belschak F.D. (2012). Work engagement and Machiavellianism in the ethical leadership process. J. Bus. Ethics.

[27] Sung M., Yoon D.Y., Han C.S.H. (2022). Does job autonomy affect job engagement? Psychological meaningfulness as a mediator. Soc. Behav. Personal. Int. J..

[28] Sekiguchi T., Li J., Hosomi M. (2017). Predicting job crafting from the socially embedded perspective: The interactive effect of job autonomy, social skill, and employee status. J. Appl. Behav. Sci..

[29] Gagné M., Tian A.W., Soo C., Zhang B., Ho K.S.B., Hosszu K. (2019). Different motivations for knowledge sharing and hiding: The role of motivating work design. J. Organ. Behav..

[30] Lips-Wiersma M., Morris L. (2009). Discriminating between ‘meaningful work’ and the ‘management of meaning’. J. Bus. Ethics.

[31] Chalofsky N. (2003). An emerging construct for meaningful work. Hum. Resour. Dev. Int..

[32] Dai G., Ferry K., Spencer S., Blazek S., Lirio (2021). Purpose and Purposefulness at Work: The Impact on Employee Engagement and Organizational Commitment. J. Organ. Psychol..

[33] Johns G., Xie J.L., Fang Y. (1992). Mediating and moderating effects in job design. J. Manag..

[34] Negri L., Cilia S., Falautano M., Grobberio M., Niccolai C., Pattini M., Pietrolongo E., Quartuccio M.E., Viterbo R.G., Allegri B. (2022). Job satisfaction among physicians and nurses involved in the management of multiple sclerosis: The role of happiness and meaning at work. Neurol. Sci..

[35] Allan B.A., Batz-Barbarich C., Sterling H.M., Tay L. (2019). Outcomes of meaningful work: A meta-analysis. J. Manag. Stud..

[36] Crawford W.S., Thompson M.J., Ashforth B.E. (2019). Work-life events theory: Making sense of shock events in dual-earner couples. Acad. Manag. Rev..

[37] Hamama-Raz Y., Hamama L., Pat-Horenczyk R., Stokar Y.N., Zilberstein T., Bron-Harlev E. (2021). Posttraumatic growth and burnout in pediatric nurses: The mediating role of secondary traumatization and the moderating role of meaning in work. Stress Health.

[38] Wrzesniewski A., Dutton J.E. (2001). Crafting a job: Revisioning employees as active crafters of their work. Acad. Manag. Rev..

[39] Berg J.M., Grant A.M., Johnson V. (2010). When callings are calling: Crafting work and leisure in pursuit of unanswered occupational callings. Organ. Sci..

[40] Zhang F., Parker S.K. (2019). Reorienting job crafting research: A hierarchical structure of job crafting concepts and integrative review. J. Organ. Behav..

[41] Tims M., Bakker A.B., Derks D. (2012). Development and validation of the job crafting scale. J. Vocat. Behav..

[42] Ghitulescu B.E. (2006). Shaping Tasks and Relationships at Work: Examining the Antecedents and Consequences of Employee Job Crafting. Ph.D. Thesis.

[43] Zhang F., Parker S.K. (2022). Reducing demands or optimizing demands? Effects of cognitive appraisal and autonomy on job crafting to change one’s work demands. Eur. J. Work Organ. Psychol..

[44] Higgins E.T. (1998). Promotion and prevention: Regulatory focus as a motivational principle. Adv. Exp. Soc. Psychol..

[45] Parker S.K., Bindl U.K., Strauss K. (2010). Making things happen: A model of proactive motivation. J. Manag..

[46] Griffin M.A., Neal A., Parker S.K. (2007). A new model of work role performance: Positive behavior in uncertain and interdependent contexts. Acad. Manag. J..

[47] Lyons P. (2008). The crafting of jobs and individual differences. J. Bus. Psychol..

[48] Debus M.E., Gross C., Kleinmann M. (2020). The power of doing: How job crafting transmits the beneficial impact of autonomy among overqualified employees. J. Bus. Psychol..

[49] Frijda N.H., Mesquita B., Sonnemans J., Van Goozen S. (1991). The duration of affective phenomena or emotions, sentiments and passions. International Review of Studies on Emotion.

[50] Houlfort N., Vallerand R. (2003). Passion at work: Toward a new conceptualization. Social Issues in Management.

[51] Seguin-Levesque C., Lalibertea M.L.N., Pelletier L.G., Blanchard C., Vallerand R.J. (2003). Harmonious and obsessive passion for the Internet: Their Associations with the Couple’s relationship 1. J. Appl. Soc. Psychol..

[52] Zigarmi D., Nimon K., Houson D., Witt D., Diehl J. (2011). A preliminary field test of an employee work passion model. Hum. Resour. Dev. Q..

[53] Amarnani R.K., Lajom J.A.L., Restubog S.L.D., Capezio A. (2020). Consumed by obsession: Career adaptability resources and the performance consequences of obsessive passion and harmonious passion for work. Hum. Relat..

[54] Jan G., Zainal S.R.M., Lee M.C.C. (2021). HRM practices and innovative work behavior within the hotel industry in Pakistan: Harmonious passion as a mediator. J. Hum. Resour. Hosp. Tour..

[55] Ho V.T., Wong S.S., Lee C.H. (2011). A tale of passion: Linking job passion and cognitive engagement to employee work performance. J. Manag. Stud..

[56] István T.K., Beáta B., Éva G., Gábor O., Adrien R. (2021). Perceived Parenting Practices as Predictors of Harmonious and Obsessive Passion Among High Schoolers and Adults. J. Happiness Stud..

[57] Wojtczuk-Turek A. (2016). Duty, Calling or Passion? The Meaningfulness of Work in Narratives of Public Administration Employees. Probl. Zarządzania.

[58] Slemp G.R., Zhao Y., Hou H., Vallerand R.J. (2021). Job crafting, leader autonomy support, and passion for work: Testing a model in Australia and China. Motiv. Emot..

[59] Fredrickson B. (2001). The role of positive emotions in positive psychology. The broaden-and-build theory of positive emotions. Am. Psychol..

[60] Ho V.T., Kong D.T., Lee C.H., Dubreuil P., Forest J. (2018). Promoting harmonious work passion among unmotivated employees: A two-nation investigation of the compensatory function of cooperative psychological climate. J. Vocat. Behav..

[61] Hannan M.T., Freeman J. (1984). Structural inertia and organizational change. American Sociological Review.

[62] Tsoukas H., Chia R. (2002). On organizational becoming: Rethinking organizational change. Organ. Sci..

[63] Dina E.C., Popescu D.V., Cazacu M. (2022). The Impact of the Employeesâ€™ Perception from the Educational Institutions in the Process of Organizational Change and the Implications of the Change on the Performance of the Organization. Ovidius Univ. Ann. Econ. Sci. Ser..

[64] Fugate M., Kinicki A.J., Prussia G.E. (2008). Employee coping with organizational change: An examination of alternative theoretical perspectives and models. Pers. Psychol..

[65] Raza M.A., Khan M.M., Mujtaba B.G. (2018). The impact of organizational change on employee turnover intention: Does stress play a mediating role?. Public Organ. Rev..

[66] Mühlemann N.S., Steffens N.K., Ullrich J., Haslam S.A., Jonas K. (2022). Understanding responses to an organizational takeover: Introducing the social identity model of organizational change. J. Personal. Soc. Psychol..

[67] Chih W.H.W., Yang F.H., Chang C.K. (2012). The study of the antecedents and outcomes of attitude toward organizational change. Public Pers. Manag..

[68] Lau C.M., McMahan G.C., Woodman R.W. (1996). An international comparison of organization development practices the USA and Hong Kong. J. Organ. Chang. Manag..

[69] Vallerand R.J. (2015). The Psychology of Passion: A Dualistic Model.

[70] Wisse B., Sleebos E. (2016). When change causes stress: Effects of self-construal and change consequences. J. Bus. Psychol..

[71] Cardon M.S., Wincent J., Singh J., Drnovsek M. (2009). The nature and experience of entrepreneurial passion. Acad. Manag. Rev..

[72] Groves R.M., Fowler F.J., Couper M.P., Lepkowski J.M., Singer E., Tourangeau R. (2011). Survey Methodology.

[73] De Beuckelaer A., Lievens F. (2009). Measurement Equivalence of Paper-and-Pencil and Internet Organisational Surveys: A Large Scale Examination in 16 Countries. Appl. Psychol..

[74] Kirmeyer S.L., Shirom A. (1986). Perceived job autonomy in the manufacturing sector: Effects of unions, gender, and substantive complexity. Acad. Manag. J..

[75] Bunderson J.S., Thompson J.A. (2009). The call of the wild: Zookeepers, callings, and the double-edged sword of deeply meaningful work. Adm. Sci. Q..

[76] Vallerand R.J., Blanchard C., Mageau G.A., Koestner R., Ratelle C., Léonard M., Gagné M., Marsolais J. (2003). Les passions de l’ame: On obsessive and harmonious passion. J. Personal. Soc. Psychol..

[77] Berg J.M., Dutton J.E. (2008). Crafting a Fulfilling Job: Bringing Passion into Work.

[78] Petrou P., Demerouti E., Peeters M.C., Schaufeli W.B., Hetland J. (2012). Crafting a job on a daily basis: Contextual correlates and the link to work engagement. J. Organ. Behav..

[79] Rafferty E., Griffin A. (2006). Perceptions of organizational change: A stress and coping perspective. J. Appl. Psychol..

[80] Baron R.M., Kenny D.A. (1986). The moderator-mediator variable distinction in social psychological research: Conceptual, strategic, and statistical considerations. J. Personal. Soc. Psychol..

[81] Omar E.N., Hassan N., Demong N.A.R., Hassan L.F.A., Alwi A. (2022). The Influence of Job Characteristics towards Job Outcomes among the Employees. Glob. Bus. Manag. Res..

[82] Park C. (2010). Making sense of the meaning literature: An integrative review of meaning making and its effects on adjustment to stressful life events. Psychol. Bull..

[83] Deci L., Ryan M. (2000). The what and why of goal pursuits: Human needs and the self-determination of behavior. Psychol. Inq..

[84] Bommer W.H., Rich G.A., Rubin R.S. (2005). Changing attitudes about change: Longitudinal effects of transformational leader behavior on employee cynicism about organizational change. J. Organ. Behav. Int. J. Ind. Occup. Organ. Psychol. Behav..

[85] Cullen-Lester K.L., Webster B.D., Edwards B.D., Braddy P.W. (2019). The effect of multiple negative, neutral, and positive organizational changes. Eur. J. Work Organ. Psychol..

[86] Rengkung L.R. (2022). Exploration and Exploitation: Driving Organizational Capability and Organizational Change Toward Competitive Advantage. Manag. Theory Stud. Rural Bus. Infrastruct. Dev..

[87] Hommelhoff S., Weseler D., Niessen C. (2021). The role of cognitive job crafting in the relationship between turnover intentions, negative affect, and task mastery. Anxiety Stress. Coping.

[88] Loi R., Xu A.J., Chow C.W., Chan W.W. (2020). Linking customer participation to service employees’ work-to-family enrichment: The role of job crafting and OBSE. J. Occup. Organ. Psychol..

